# Prey Patch Patterns Predict Habitat Use by Top Marine Predators with Diverse Foraging Strategies

**DOI:** 10.1371/journal.pone.0053348

**Published:** 2013-01-03

**Authors:** Kelly J. Benoit-Bird, Brian C. Battaile, Scott A. Heppell, Brian Hoover, David Irons, Nathan Jones, Kathy J. Kuletz, Chad A. Nordstrom, Rosana Paredes, Robert M. Suryan, Chad M. Waluk, Andrew W. Trites

**Affiliations:** 1 College of Earth, Ocean, and Atmospheric Sciences, Oregon State University, Corvallis, Oregon, United States of America; 2 Department of Fisheries and Wildlife, Oregon State University, Corvallis, Oregon, United States of America; 3 Marine Mammal Research Unit, Fisheries Centre, and Department of Zoology, University of British Columbia, Vancouver, Canada; 4 Moss Landing Marine Laboratories, University of California, Moss Landing, California, United States of America; 5 US Fish and Wildlife Service, Anchorage, Alaska, United States of America; National Oceanic and Atmospheric Administration/National Marine Fisheries Service/Southwest Fisheries Science Center, United States of America

## Abstract

Spatial coherence between predators and prey has rarely been observed in pelagic marine ecosystems. We used measures of the environment, prey abundance, prey quality, and prey distribution to explain the observed distributions of three co-occurring predator species breeding on islands in the southeastern Bering Sea: black-legged kittiwakes (*Rissa tridactyla*), thick-billed murres (*Uria lomvia)*, and northern fur seals (*Callorhinus ursinus*). Predictions of statistical models were tested using movement patterns obtained from satellite-tracked individual animals. With the most commonly used measures to quantify prey distributions - areal biomass, density, and numerical abundance - we were unable to find a spatial relationship between predators and their prey. We instead found that habitat use by all three predators was predicted most strongly by prey patch characteristics such as depth and local density within spatial aggregations. Additional prey patch characteristics and physical habitat also contributed significantly to characterizing predator patterns. Our results indicate that the small-scale prey patch characteristics are critical to how predators perceive the quality of their food supply and the mechanisms they use to exploit it, regardless of time of day, sampling year, or source colony. The three focal predator species had different constraints and employed different foraging strategies – a shallow diver that makes trips of moderate distance (kittiwakes), a deep diver that makes trip of short distances (murres), and a deep diver that makes extensive trips (fur seals). However, all three were similarly linked by patchiness of prey rather than by the distribution of overall biomass. This supports the hypothesis that patchiness may be critical for understanding predator-prey relationships in pelagic marine systems more generally.

## Introduction

Predators and their prey must overlap in space and time for predators to survive [1], [2]. Despite the complex behavioral interactions observed between predators and their prey [3], [4], [5], the expected coherence between predators and their prey is commonly observed in terrestrial [6], [7], [8], aquatic, [9], [10], and benthic marine systems [11], [12], [13], [14]. Yet, in marine pelagic systems, many studies have found weak or ephemeral spatial associations between predators and pelagic prey ([15], [16], [17], [18] but see [19], [20], [21], [22], [23], [24], [25], [26], [27]) and even negative relationships [28], resulting in a large number of novel hypotheses to explain the divergence. Often, our measures of pelagic prey distributions have thus not helped us to understand a central issue in ecology, the mechanisms underlying the distribution of predators in their habitat [29].

Studies of marine predator-prey relationships most often examine the spatial relationship between the species of interest and the biomass or abundance of its prey spatially integrated or averaged in some way (e.g. g/m^2^ or individuals/m^2^ over some prescribed transect length) [30], [31], [32], [33]. However, a ubiquitous feature of pelagic marine systems is the spatial aggregation of resources, or patchiness [34]. As a result, concentrations of prey rarely occur within the habitat at the average levels we typically describe. For example, if a 100 m long, 10 m high school of fish with a numerical density of 100 fish per m^3^ was found over a bottom depth of 100 m, various areal density estimates are made as the integration scale changes: 100 m = 10 fish m^−2^; 1 km = 1 fish m^−2^, 10 km = 0.1 fish m^−2^. A predator, however, is able to find one of two states, a school at a density of 100 fish m^−3^, or empty water. Attempts have been to deal with this kind of spatial variability in prey primarily using approaches that identify scale-specific relationships (e.g. [35], [36]), assuming that the scales of prey patches are consistent. If, however, patches occur over a gradient of sizes and densities, or predators do not choose prey based on patch size, correlations between predator and prey remain obscured.

In order to understand predator distributions, we must characterize prey in a way that is relevant to the costs and benefits for each predator species, its *suitability* [2]. While the abundance or biomass of prey is one consideration, the distribution of prey has been shown to have a strong effect on the energetic gains and costs of foraging [37], foraging success, and overall predator performance [38] while the spatial scales of prey aggregation can affect the ability of a predator to detect and remain in a prey patch [39] and prey density and its variability determine predator consumption rate and efficiency [40], [41], [42]. The goal of this work was to use measures of the environment, prey abundance, prey quality, and prey distribution to identify the key features of prey suitability that predict the distributions of three co-occurring predator species breeding on islands in the southeastern Bering Sea: black-legged kittiwakes (*Rissa tridactyla*), thick-billed murres (*Uria lomvia)*, and northern fur seals (*Callorhinus ursinus*). To accomplish this goal, we combined visual surveys of predators with concomitant fine scale prey characterization and in situ measurements of the environment to create statistical models designed to predict predator distributions. We then tested these models using movement patterns obtained from tagged individual animals from all three species. The relative importance of the prey features identified provides insight into the foraging mechanisms used by a shallow diver that makes trips of moderate distance (kittiwakes), a deep diver that makes trip of short distances (murres), and a deep diver that makes extensive trips (fur seals).

## Methods

### Study System

As part of a large, interdisciplinary ecosystem study known as the Bering Sea Project [43], we examined the distribution and behavior of three predators breeding on islands in the southeastern Bering Sea: black-legged kittiwakes, thick-billed murres, and northern fur seals. The eastern Bering Sea shelf is a highly productive ecosystem [44]. St. George and St. Paul islands, part of the Pribilof Archipelago, sit at the edge of this shelf, providing nesting habitat for one of the largest concentrations of seabirds in the North Pacific [45] and hosting most of the world’s breeding population of northern fur seals. Bogoslof Island, recently re-colonized by fur seals, lies north of the Aleutian Archipelago adjacent to Aleutian passes and is surrounded by deep oceanic water [46]. Populations at the Pribilof Islands and Bogoslof Island and are undergoing different trajectories with numbers of seabirds and fur seals declining on St. Paul Island, stable at St. George Island, and increasing at Bogoslof Island [47], [48].

On all three islands, adults of the three focal species are central place foragers that are constrained in foraging distance and duration by the fasting abilities of their offspring [49] which are provisioned by their parents during July and August. These three species, however, have very different foraging strategies and constraints. Black-legged kittiwakes feed at or near the surface on pelagic fish and invertebrates. They are efficient flyers that can make relatively long trips, with both male and females delivering multiple prey items stored in their crop to their chicks [50]. Thick-billed murres are pursuit-diving predators that feed on a variety of fish, zooplankton, and other invertebrate prey [51] at depths up to 200 m [52], [53]. Murres have high energetic flight costs and thus make relatively short foraging trips; adults of both sexes return to provision their chicks with individual fish and squid brought to the breeding site in the bill, often prey that is larger than what they themselves eat [51]. Fur seals feed mainly on juvenile walleye pollock (*Theragra chalcogramma*), squid, and vertically migrating mesopelagic fish, diving to depths of up to 200 m to forage [54]. They supply milk to their young during periodic haul outs between extensive feeding trips that last days and cover distances of up to 200 km from the rookeries [55]. Together, these three focal predator species cover a wide range of central place foraging strategies.

### Approach

Data were collected from mid-July to mid-August of 2008 around the Pribilof Islands and during the same time period in 2009 around both the Pribilof Islands and the adjacent area around Bogoslof Island. Ship-based sampling of the environment, potential prey, and the density of birds and fur seals was conducted in a 200 km radius around each colony along 289, 10-km long transects that were placed in a random design stratified amongst three topographic zones. These data were used to create a statistical model predicting the observed predator spatial distributions which was then tested against data collected on habitat use by individual predators tagged at the colonies during the same time period. Detailed methods for the ship-based sampling, summarized here, are included in Benoit-Bird et al. [27].

### Ethics Statement

All research was conducted in accordance with the Animal Care and Use Committees of the respective institutions of the author responsible for those data and complied with all applicable laws. Vertebrate prey data was collected in accordance with the American Fisheries Society’s Guidelines for the Care and Use of Fish in Research and the Institutional Animal Care and Use Committee of Oregon State University (permit 3659). Fur seals, covered by the US Marine Mammal Protection Act, were studied under the National Oceanographic and Atmospheric Administration (NOAA) permit number 14329 and abided by the guidelines of the Committee on Animal Care at the University of British Columbia (permit A09-0345). Seabird cliffs on the Pribilof Islands are part of the Alaska Maritime National Wildlife Refuge. Seabirds were studied in a collaborative effort with Refuge staff (permit 20570), following the United States Government Principles for the Utilization and Care off Vertebrate Animals and the Animal Care Committee of the United States Fish and Wildlife Service (permit 200908). Access to Bogoslof Island, part of the National Wilderness Preservation System, was granted by the United States Fish and Wildlife Service.

### Physical and Biological Environment

Environmental data were collected using a CTD (conductivity, temperature, depth) equipped with a fluorometer and dissolved oxygen sensor that was profiled to 100 m, or 5 m above the seafloor, whichever was shallower, at the beginning of each transect. From these data, a variety of physical habitat measures were calculated including sea surface temperature (°C), sea surface salinity, thermocline depth (m), mean temperature above the thermocline (°C), mean temperature below the thermocline (°C), oxycline depth (m), minimum oxygen saturation (%), minimum oxygen saturation depth (m), water column stratification (σ_t_/m), stratification above the thermocline (σ_t_/m), and stratification below the thermocline (σ_t_/m),. Biological habitat was characterized using vertically integrated chlorophyll concentration (mg/m^2^), the chlorophyll maximum (mg/m^3^), and the depth of the chlorophyll maximum (m). A tow vertically integrated to 100 m for meso-zooplankton conducted at the beginning of each transect was used to measure meso-zooplankton biomass (g/m^2^). Additional environmental variables included range of the transect from the nearest colony (km) and seafloor depth (m) derived from acoustic measurements (<1000 m) or charts (>1000 m).

### Prey Fields

A single, depth-targeted net trawl for nekton and macro-zooplankton (e.g. krill) was conducted on each transect using an 8 m by 8 m opening Marinovich midwater trawl fitted with a 3 mm cod-end mesh liner. These data were used to measure the identity, size, weight and proximate composition of potential prey. The median individual length for each major group (mm) and average energy content per prey (kJ/individual) [56] were used in the statistical model as measures of individual prey characteristics. For taxonomic groups including squid, amphipods, vertically migrating mesopelagic fish, and epipelagic fish (excluding pollock) that were relatively rare and could not be enumerated with acoustics, relative abundance across the study area was calculated using the trawl data. Absences were used as one class while positive counts of each taxon were grouped into quartiles of abundance for a total of five abundance classes for each taxonomic group.

Euphausiids (*Thysanoessa* spp.) and fish (overwhelmingly dominated by juvenile walleye pollock in their first two years of life) were identified using frequency differencing of the acoustical scattering data [57] as in [27]. All data not matching the “fish” characteristics were removed from the raw 38 kHz echogram and all data not matching the “euphausiid” characteristics were removed raw 120 kHz echogram for additional prey analyses. Data from each frequency were then thresholded to −80 dB re 1 m^−1^ to remove weak scattering and noise and integrated over 200 m long sections along each transect. These values were combined with measurements of median individual length and wet weight along with published acoustic relationships to these measurements (pollock: [58], [59]; euphausiids: [60], [61]) to estimate the average abundance (individuals/m^2^) and biomass (g/m^2^; often referred to as ‘biomass density’ or simply ‘density’) of pollock and euphausiids as well as the variance in each of these measures for each transect over the full 100 m depth range and in 20 m depth slices. Juvenile pollock (“fish”) data were grouped as a single class as well as being apportioned to either young of the year (age-0) or age-1 pollock using trawl data. Since only two transects contained both age classes and both of these were overwhelmingly dominated by a single year class, we used simple proportions weighted by the relative length difference of the two classes to apportion age classes.

Euphausiids and pollock were both observed to be highly spatially aggregated so that all transects on which these species were detected contained at least one discrete patch [27]. Myriax’s Echoview Software, School Detection module was used to identify aggregations of each taxon within the masked, full-resolution echograms. Simply, this approach looks for a minimum number of contiguous values in both the distance and depth directions above a set threshold [62]. For euphausiids, masked 120 kHz data must have been greater than −75 dB re 1 m^−1^ for at least 1 m vertically and 5 m along track as corrected for beam effects [63], resulting in more than 95% of pixels classified as “euphausiids” to be encompassed in patches. For pollock, spatial distributions were determined to be hierarchically distributed with dense, ovoid patches inside larger, more loosely aggregated layers [64]. As a result, two data thresholds were utilized; a relatively low threshold of −65 dB re 1 m^−1^, which equated to 0.1 fish/m^3^ for median sized pollock and encompassed more than 97% of all data classified as pollock, and −59 dB re 1 m^−1^, which equated to 0.5 fish/m^3^ for the same sized fish. Experimentation with this higher threshold showed no significant change in the mean volume scattering strength measured within each patch or the horizontal and vertical size of each patch with thresholds between −62 and −53 dB re 1 m^−1^ despite changes in the number of patches detected, indicating the high contrast between these patches and the remainder of pollock. The threshold of −59 dB was chosen to maximize the number of transects with detected patches while detecting patches that were quantified to have ovoid (“school”-like) rather than amoeba-like shapes using the same contiguity size minimums used for euphausiid patches.

Prey spatial distribution characteristics were measured for each surveyed transect on which prey were detected (N_pollock_ = 221, N_euphausiids_ = 247, N_total_ = 289). The minimum, maximum, and median depth of pollock and euphausiids along each transect were calculated. For aggregations of euphausiids and both aggregation classes of pollock, the mean aggregation horizontal size, height, area, and distance to nearest neighboring aggregation of the same type were measured for each transect. Data within the identified boundaries of each aggregation were then thresholded at a value of −85 dB re 1 m^−1^ before the data were integrated over the patch area to provide mean volume backscattering which was converted to density of individuals (fish/m^3^ or euphausiids/m^3^) using echo energy integration [65]. The minimum, maximum, and mean numerical densities within aggregations were calculated over the upper 100 m of the water column and in 20 m vertical slices for each transect. In addition, the average number of individuals per aggregation and the density of aggregations along a transect (patches/km^2^) were estimated. The biomass (g/m^2^) and numerical abundance (fish/m^2^) of pollock in dense aggregations as well as the proportion of these measures relative to all pollock were estimated along with the density of these dense aggregations on each transect (patches/km^2^). The minimum, maximum, and median depth of these dense patches was then measured. The relationships between the biomass and numerical abundance of prey to patch characteristics were explored using regression analysis.

### Explaining Observed Predator Distributions

Visual surveys for birds and mammals were conducted by a single observer 6 m above the waterline from the starboard side of the vessel’s wheelhouse over the entire length of each transect. Surveys employed a strip transect technique consistent with historic surveys in the North Pacific and Bering Seas [66], [67], [68], providing densities for three focal predator species on the surface of the water out to 300 m from one side of the vessel. These observations were used as the dependent measure in multiple-regression models incorporating measured habitat and prey descriptors. The goal of this was not just to create a statistical model that matched the observations but rather to use these data to parameterize a predictive model that could be used to identify patterns in relationships identified as biologically relevant, and subsequently test this model with novel data. Although more complex statistical approaches like generalized additive models or environmental envelopes might improve the statistical fit of the observations, relationships among variables are difficult to interpret using these approaches and our fundamental understanding of the mechanisms underlying observed relationships is often obscured by the increased complexity [29].

Best subsets multiple linear regression model selections using Akaike’s information criteria (AIC_C_) for inclusion of explanatory variables were performed on the 173 transects (of 289) for which visual observations were available. Before analysis, each variable was assessed for normality and homoscedasticity, transformed as appropriate, and outliers (Cook’s D>0.025) trimmed. A correlation matrix showed that none of the transformed explanatory variables demonstrated correlation values greater than 0.75, suggesting limited collinearity [69]. To further minimize the effects of collinearity, explanatory variables with tolerance values of less than 0.10 were not allowed in the final model. Any variables with low tolerances were tested independently to determine which were the most significant within the model and only the strongest variable was retained in the final model. The effect of sampling year on the fit of the model to the data was tested by running the full model for each predator separately for each year. An F statistic was then used to test for changes in R^2^ values. To examine the effect of colony on the fit of the model to the data, transects were broken up by their location into three, non-independent groups, each based on a circle with a radius of 100 km centered on each island, an approximation of the foraging arena for each colony/rookery. An F statistic was used to test for changes in the resulting R^2^ values.

In addition to model selection employing all available explanatory variables, model selection was run on subsets of these variables to determine the relative contribution of different measure types including all variables that describe the environment (17), all variables that describe individual prey characteristics (12), all variables that describe prey abundance or biomass (50), and all variables that describe the spatial distribution of prey (48 e.g. depth, patch size, prey density within a patch). In order to test the hypothesis about what types of prey characteristics were most important in determining predator distributions, all variables that have previously been hypothesized to affect these predators and the new spatial distribution parameters suggested here were included in the full model. While this meant a large number of variables (127), it provided the greatest possible fit between the model and the data which allowed decrements in fit in the subsets model to be easily observed, testing the hypothesis about prey characteristics. The four subsets models were each run independently and then each prey subset type was run with the environmental descriptors to facilitate comparisons with previously published work.

The full models for each predator were used to create predator density predictions for each species in each sampling year. In both years, approximately 60% of transects had visual survey data that were used to generate the statistical model. The remaining 40% of transects did not include visual surveys because they were surveyed at night (85%) or when fog or weather limited visibility, yet these transects had prey and oceanographic data. Therefore, all were used to create predicted predator distributions.

### Assessing Predicted Predator Distributions

The predator distributions predicted by the best full subsets models were tested against habitat used by individually tracked predators of all three species. Individual, breeding adults from each of the three focal species on each colony were fitted with archival tags that recorded both their position and some measure of activity indicative of foraging behavior for the species. Full methods for each species can be found elsewhere but key details are summarized here.

In 2009, lactating female seals were tagged at St. Paul Island (N = 16) and Bogoslof Island (N = 21) using archival GPS tags that carried tri-axial accelerometers and magnetometers and a depth sensor [70]. Using the 1-s resolution reconstructed locations between known GPS fixes, areas of fur seal retention were identified using patterns in the tortuosity, a measure of the linearity of an animal’s swimming path. Wherever a 10-point running measure of tortuosity of an individual track was in the upper quartile of all measured tortuosity values for at least five points in a row, the location was identified as a foraging patch. If these patches were less than 100 m from a neighboring patch, they were grouped together as a foraging area. Each area was then weighted by the amount of time spent within it.

During 2009, 10 thick-billed murres from St. George Island and 14 from Bogoslof Island were fitted with time-depth recorders and GPS loggers [71]. Tagged birds were rearing chicks 5–15 days old. Tags recorded position and depth at 1 to 2 second intervals. The locations of dives greater than 7 m, the minimum consistently recorded depth, were identified as potential foraging locations.

Between 13 and 15 adult black-legged kittiwakes raising chicks were tagged at each colony in each year: both Pribilof Islands in 2008 and the Pribilof Islands and Bogoslof Island in 2009 [71]. Each individual was fitted with GPS and activity loggers that provided location and wetness at 1–120 s intervals over 2–15 day periods. Following Paredes et al [71], presumed foraging events were identified whenever the tag was both wet and dry during a 10 minute interval.

For each predator species, during the periods that overlapped with the ship-based sampling effort, locations where tagged animal behavior was consistent with foraging were used to generate horizontal habitat-use kernels for all tracked individuals from each island. These adaptive kernels were optimized by least-squares cross validation [72] using analysis grid cells of 100 m and smoothed at a scale of 20 km, double the length of the survey transects. For each species on each island, the 95% utilization contour was used to define the foraging arena, the 75% kernel higher foraging effort areas, and the 50% kernels the core foraging area. These kernels were then combined for comparison to model predictions by choosing the highest use descriptor kernel (95%, 75, or 50%) for each species at the location of the midpoint of each transect from the survey.

Predator densities predicted by the models were binned into four classes – one class for no predators, and three classes based on the distribution of the positive density values delineated by the 5th percentile, 25th percentile, and 50th percentile. These classes were chosen to approximate the distributions described for tagged animals using the 95%, 75%, and 50% kernel density distributions. The predicted classification from the statistical model and the kernel density classifications from the tagged were compared for each predator using Wilcoxon Signed Ranks tests. A chi-squared test was used to examine the distribution of the difference between the model prediction and the density distribution kernels of the tagged animals of each species. Distance from the nearest colony/rookery was grouped by 25 km increments into five classes (% of total transects): <25 km (9%), 25–50 km (16%), 50–75 km (10%), 75–100 km (9%), 100–150 km (28%), and >150 km (28%). The effect of these distance classes on the difference between the model predictions and the kernels of the tagged animals was examined with contingency analysis.

To determine if the fit between the modeled predator distributions and the distributions of tagged animals was different across species, a Kruskal-Wallis test was used to assess the effect of species on the difference between model and kernel categories. Only data over the distance class range for each species that was determined not to be significantly biased was included in the analysis.

## Results

**Figure 1:**
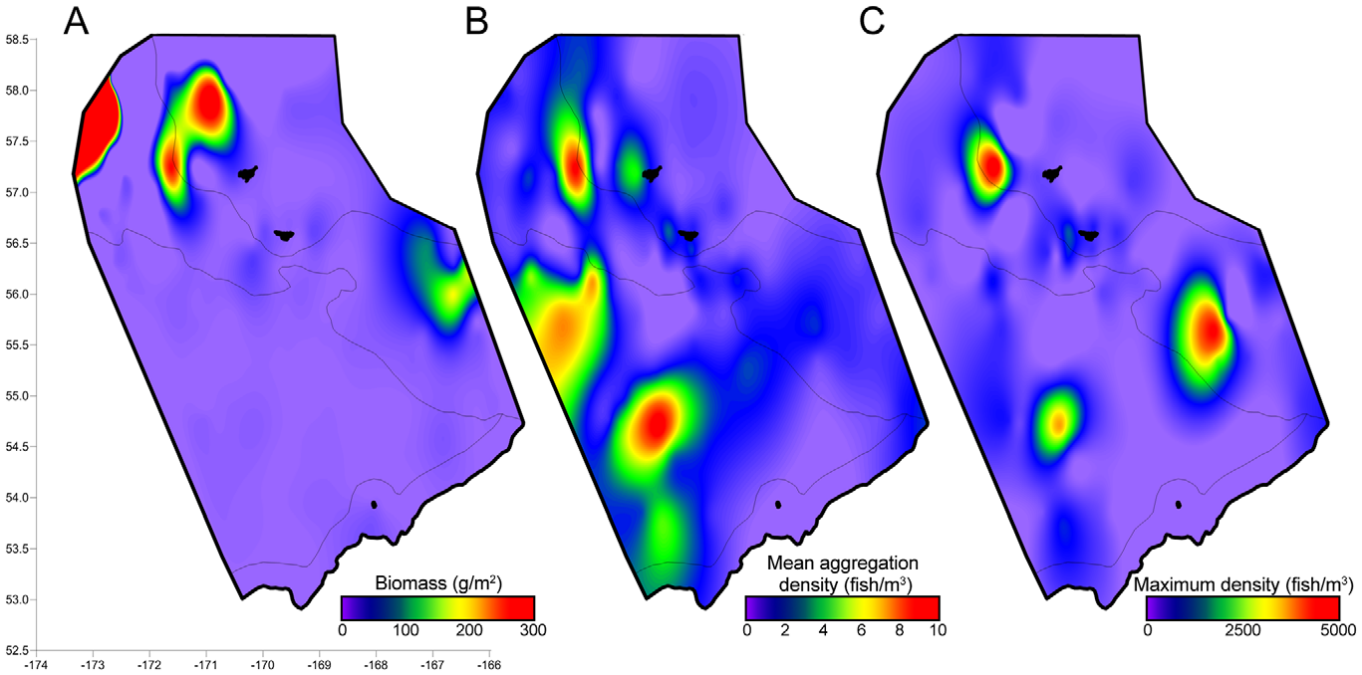
The distribution of juvenile walleye pollock in 2009 based on three different metrics. A. biomass density, the most commonly used measure, B. the mean volumetric density of pollock within aggregations, a measure of local density within a patch, and C. the maximum volumetric density of pollock per sampling transect. Map surfaces were generated using minimum curvature interpolations (N = 165).

**Figure 2:**
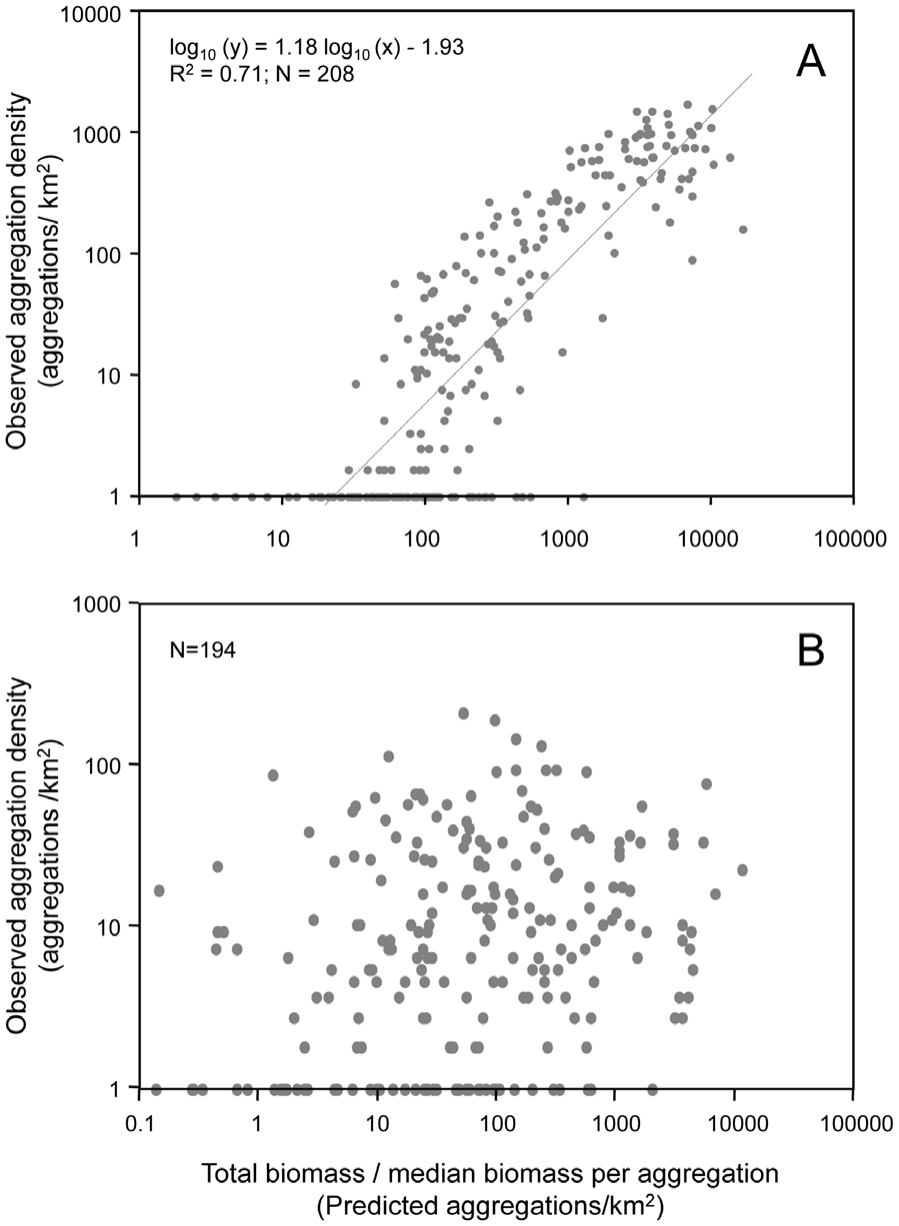
The observed versus expected density of prey aggregations on each transect. A. Shows dense pollock aggregations and B. euphausiid aggregations. Only transects on which these groups were detected were included. The expected density of aggregations is the total biomass for each transect divided by the median biomass per aggregation observed across all transects. Note that in panel A there are 23 data points to the left of the regression line on the x axis but because of overlapping values, it is not possible to see each point.

The spatial distribution of prey varies dramatically depending on the metric utilized to characterize the prey field, illustrated for pollock in Figure 1. Despite the fact that each of these metrics has been proposed as a simple way to characterize the prey field, these metrics showed little relationship to each other. There were no significant relationships between the biomass or abundance of prey and the density of prey in a patch, patch size measures, or patch spacing (p>>0.05 for all comparisons), making it impossible to convert from one metric to another. There was, however, a significant positive relationship between the biomass of pollock on a transect, quantified as the number of aggregations expected, and the detection of aggregations of pollock (Figure 2a; R^2^ = 0.71, p<0.001). However, there was a threshold biomass value above which pollock began to form aggregations; below this threshold, only scattered individual pollock were identified. This aggregation threshold was roughly 10 times the median biomass observed for a single aggregation of pollock or approximately 100 times the biomass of the minimum pollock aggregation (quantified as the 5^th^ percentile of all observed aggregations, not shown). In contrast, no pattern between biomass and density of aggregations was observed for euphausiids (Figure 2b).

**Figure 3:**
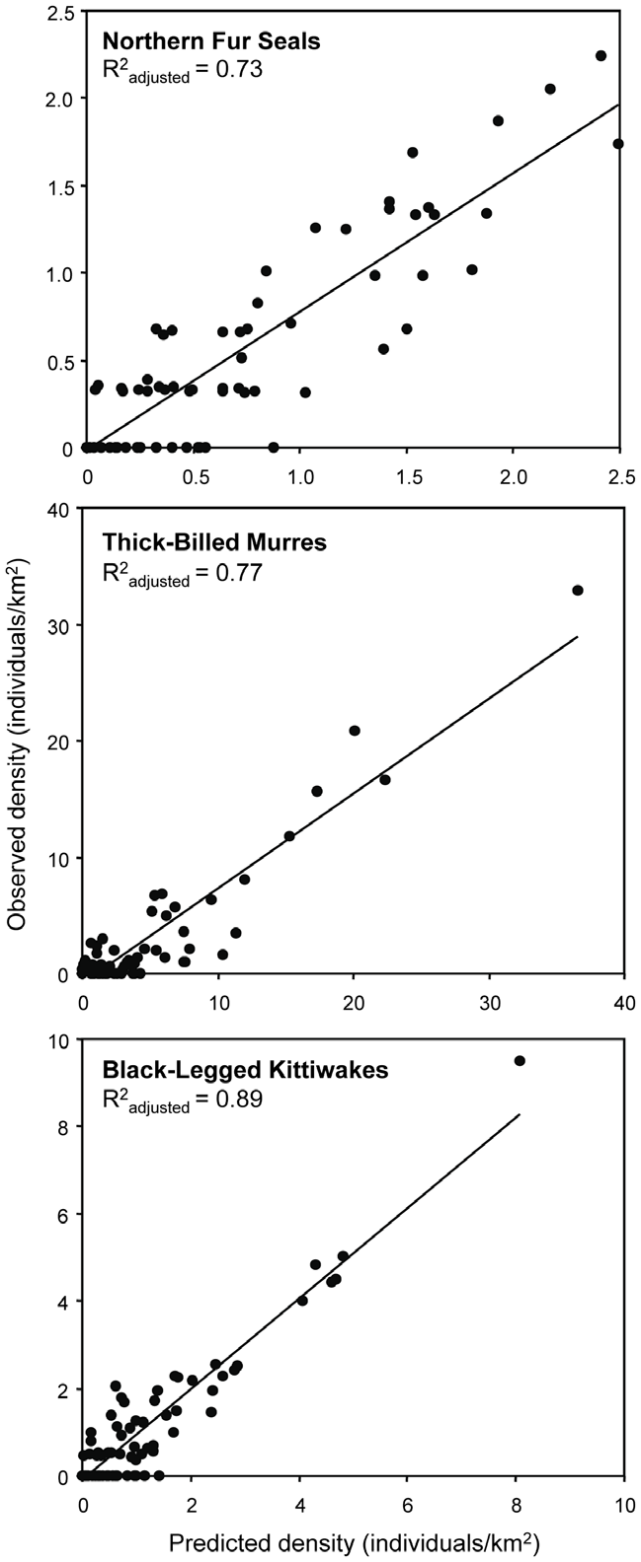
Predicted and observed predator densities. The observed density of each predator versus the density predicted by the full multiple regression model for each species.

All of the habitat and prey metrics were used in multiple regression modeling to explain the distribution of the three focal predator species observed using at-sea observations. The best-fit models (summarized in Table 1 with results shown in Figure 3) show that in only one species does any measure of prey abundance play a role in predicting predator distributions. For all species, the most important variables were measures of prey density and vertical distribution. For each species, a measure of euphausiid size or quality and measures of environmental structure are also important. Using the variables shown for each species to predict the distributions of predators separately for each year showed no significant change in R^2^ values for any species (F-tests, df = 1, p>>0.05 for all comparisons). Splitting the data into three, non-independent circles with radii of 100 km centered on each island, an approximation of the foraging arena for each colony/rookery, there was no significant change in R^2^ values among circles (F-tests, df = 2, p>>0.05 for all comparisons).

**Table 1:** Summary of best subsets multiple regression models for densities each of three focal predators visually surveyed in the Southeastern Bering Sea in 2008 and 2009.

Northern Fur Seals	β		Thick-Billed Murres	β		Black-Legged Kittiwakes	β	
Pollock Maximum Depth (m)	+	0.46	Pollock Minimum Depth (m)	–	0.43	Pollock Aggregation Height (m)	+	0.42
Euphausiid Length (mm)	+	0.41	Euphausiid Energy/Individual (kJ/indiv)	+	0.42	Pollock Aggregation Density 5–20 m (indiv/m^3^)	+	0.39
Bottom Depth (m)	+	0.37	Euphausiid Mean Patch Density(indiv/m^3^)	+	0.42	Temperature Below Thermocline (°C)	–	0.37
Oxycline Depth (m)	+	0.30	Euphausiid Maximum Density (indiv/m^3^)	+	0.39	Pollock Maximum Depth (m)	–	0.37
Euphausiid Maximum Density(indiv/m^3^)	+	0.26	Pollock Aggregation Density 5–20 m (indiv/m^3^)	+	0.26	Pollock Aggregation MinimumDepth (m)	–	0.36
Pollock Aggregation Density 5–20 m (indiv/m^3^)	+	0.17	Sea Surface Temperature (°C)	+	0.11	Euphausiid Energy/Individual (kJ/indiv)	+	0.27
Pollock Aggregation Spacing (m)	–	0.15				Oxycline Depth (m)	–	0.20
Stratification Above Thermocline(σ_t_/m)	–	0.11				Squid Abundance (Classified)	+	0.16
						Sea Surface Salinity	+	0.13
**Adjusted R^2^**		**0.73**			**0.77**			**0.89**

Explanatory variables are listed in descending order of importance for each species’ model. The slope of the relationship for each explanatory variable is shown along with its regression coefficient. The R^2^ for each model adjusted for the number of variables in the model is also shown.

To test for the effects of the categories of independent variables used, models were run on subsets of input variables in four different classes (Table 2). Comparison of results across variable types allows testing of hypotheses about predator foraging strategies. For all three species, descriptions of prey patches dominate in predicting predator distributions.

**Table 2:** Summary of adjusted R^2^ for multiple regression models predicting predator densities.

Environment	Prey			Predator		
Physical & Biological	Individual characters	Abundance	Patches	Fur Seals	Murres	Kittiwakes
X	X	X	X	0.73	0.77	0.89
X				0.11	0.13	0.02
	X			0.03	0.01	0.06
		X		0.02	0.00	0.02
			X	0.65	0.71	0.80
X	X			0.13	0.13	0.07
X		X		0.18	0.14	0.12
X			X	0.72	0.77	0.82

The results of the full regression model including all independent variables are shown in the first row. In addition to the full regression models, models were run using subsets of explanatory variables separated into four classes. Each class of variables was run separately, all prey classes were run together, and each prey class was run in combination with environmental variables.

**Figure 4:**
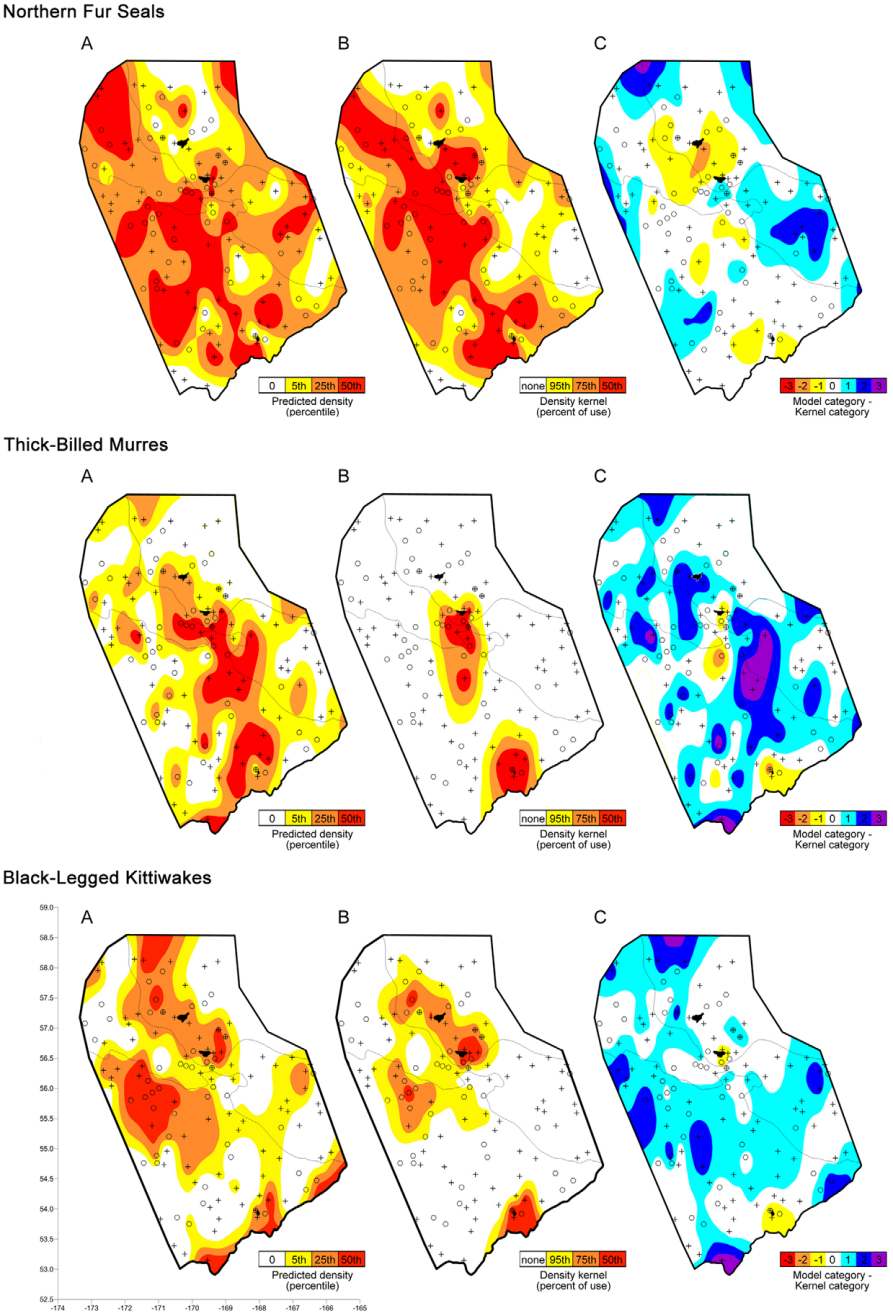
Predicted and observed predator habitat use in 2009. A. The predicted density classes for each predator species using the full multiple-regression model based on transect data and B. the kernel densities for tagged individual predators at each sampled transect. C. The difference between the model category and the kernel category. Positive, cool colored values indicate that fewer predators used an area than predicted by the model while negative, warm colored values indicate the opposite. On each plot, the center of each transect that was visually surveyed for birds and mammals and thus was used to create the regression model is shown with a +. The center of each transect for which environmental and prey data were available but could not be used to create the regression model is shown by o. Map surfaces were generated using minimum curvature interpolation that did not allow values plotted at sampled points to differ from their actual values.

The predator model categories (Figure 4a) were significantly higher than the predator tracking kernel categories (Figure 4b) for all three species (Wilcoxon Signed Ranks tests fur seals: Z = 2.04, p<0.05; murres: Z = 6.17, p<0.01; kittiwakes: Z = 6.21, p<0.01; Figure 4c); the habitat use indicated by kernels was lower than expected overall from the model predictions. Comparing the predicted to observed predator categories by looking at the difference between the two showed that the distribution of values was significantly different from random (one-sample chi-squared tests fur seals: χ^2^ = 128.9, df = 4, p<0.001: murres: χ^ 2^ = 113.9, df = 4, p<0.001; kittiwakes: χ^ 2^ = 110.5, df = 4, p<0.001; Figure 4c). For all three species, there were significantly more values where the two measures matched than expected, and significantly fewer than expected values offset by two steps or three steps than expected.

Differences between the model and the tagged predator kernels were largely explained by distance from the nearest colony. Contingency table analyses showed that at distances between 25 and 50 km from the nearest colony, tagged fur seals foraged more than expected based on model predictions while at distances greater than 150 km from the nearest colony, tagged fur seals foraged less than expected for the observed conditions; at distances less than 25 km from the nearest colony, tagged murres foraged more than expected based on the model predictions while at distances greater than 75 km from the nearest colony, tagged murres foraged less than predicted by the conditions; at distances less than 25 km from the nearest colony, tagged kittiwakes foraged more than expected from the model while at distances greater than 100 km from the nearest colony, tagged kittiwakes foraged less than expected for the conditions. When the Wilcoxon Signed Rank test was repeated for only the transects that did not show a significant bias (50–150 km for seals, 25–75 km for murres, and 25–100 km from the colonies for kittiwakes), significant differences were no longer observed between the kernels and the model (fur seals: Z = 0.62, p = 0.57; murres: Z = −0.44, p = 0.72; kittiwakes: Z = 1.01, p = 0.64).

For all three species, the availability of ship-based predator survey data had no significant effect on the agreement between the model and the kernels (ANOVA; fur seals: F = 2.66; df = 1,289; p = 0.41; β = 0.37; murres: ANOVA: F = 2.56; df = 1,289; p>0 = 0.35; β = 0.35; kittiwakes: ANOVA: F = 1.72; df = 1,289; p = 0.27; β = 0.33). There was no effect of species on the difference between model and kernel categories when each species was tested using only non-biased distance class ranges (Kruskal-Wallis χ^ 2^ = 2.01, df = 2, p>0.05).

## Discussion

We observed strong spatial coherence of three predators with their prey resources despite different foraging strategies and life history constraints. Prey variables alone explained 66–81% of the variance in predator densities observed at-sea (Figure 3, Table 1). Coherence between predators and their prey is commonly observed in terrestrial [6], [73], aquatic [9], [74], and benthic marine systems [11] but less commonly in pelagic marine systems [21], [22], [23], [24], [25], [26]. In our study, we also failed to predict at-sea predator densities when we characterized prey in terms of *areal* biomass density or numerical abundance (the approaches most commonly taken in previous efforts); as a class, these prey variables predicted an insignificant fraction of the variance in predator distributions even when variance in these measures was included. The measures most commonly used to quantify prey distributions did not reflect how these predators perceived the quality of their food supply.

For all three predators, the variables that were most important in predicting observed densities were descriptions of the distribution of prey different than those traditionally used. However, physical variables explained large proportions of the variability in the fur seal and kittiwake full models and a small proportion of the variability in the murre model. When modeled separately, however, physical variables predicted only 7–13% of the observed predator distributions. This indicates that physical habitat variables were somehow modulating the prey distributional characteristics rather than serving as indirect proxies for prey or as direct cues to the predators. Nearly all of the physical habitat variables that were identified as important in the full models were those related to vertical water column structure rather than single point measurements such as sea surface temperature. It is likely that factors such as the depth of the oxycline and the temperature below the thermocline interact to affect the vertical distribution of prey. Vertical prey structure is likely critical to the foraging success of these air-breathing predators [75], [76], [77] as it controls whether a prey item is accessible and at what cost; the vertical distribution of prey was identified as an important variable in the models for all three species of predators, as shown for murres in the study using a very different approach [27] and for breath-hold predators in other systems [76], [77]. Of note for our study is that the upper meter of the water column utilized by kittiwakes was not directly sampled yet prey variables measured deeper in the water column strongly predicted kittiwake distribution, suggesting a relationship between surface and subsurface prey features.

Diving limits may also influence how a predator exploits prey in the horizontal dimension. Because diving time is limited, the horizontal distance a predator can cover in a single dive is limited. Inter-prey spacing, measured here as *local* prey density within a patch, determines how many prey an air-breathing predator can encounter within a foraging dive. Local prey density has been shown to directly impact both immediate foraging efficiency and long-term survival in predators [40], [41] but the physical constraints of diving likely increase the importance of the relationships between local prey density and foraging efficiency. The effect of local prey density or spacing appeared to play a role at a larger scale as well in foraging fur seals. Patches of juvenile pollock, the primary food for fur seals, were quite small, with an average diameter of approximately 10 m, and these pollock patches were often clustered. Fur seals were found more often when inter-patch spacing was small, approaching the size of the patches themselves (5–10 m). This might have allowed fur seals to more efficiently access more than one pollock patch in a single dive.

Despite the importance of prey spacing for all three predators, none of the horizontal patch scale measurements were important for predicting habitat use by the predators. In other words, predators were not selecting prey based on horizontal patch size, despite a large range of patch sizes present in the habitat: 3–50 m for pollock and 5–10 km for euphausiids. Larger patches are predicted to be more conspicuous [78] and to increase a predator’s rate of prey acquisition [39]. However, because of the long transit from the nest or brood site, the importance of the selection of larger patches is greatly reduced in centrally foraging species; from an optimality perspective, “while feeding young in the nest, parents should exhibit nearly the same choice of patches whether they be large or small” [79], consistent with the observations of all three predators in our analysis. A concurrent study of the selection of individual patches by diving murres similarly showed no effect of euphausiid patch size on prey selection [27]. A direct implication of this result is that analyses focusing on scale-specific relationships between marine predators and their prey can miss the coherence between them, particularly among predators and euphausiid patches, because such patches can vary by two orders of magnitude in horizontal extent.

The spatial coherence observed between predators and their prey in our surveys was confirmed by comparing the habitat use of tagged individual predators from each colony with the distributions predicted using statistical models of the at-sea surveys. For all three predators, there was a strong fit between the models and the foraging kernels of the predators. In addition to relying on statistics that support this, it is helpful to examine specific sampling locations more closely. In particular, in evaluating predator density predictions, it is useful to look at transects where independent variables were measured but visual census data of predator densities were not available because of weather or darkness. For example, a hot spot in kittiwake foraging was predicted to the southwest of the Pribilof Islands, just off the shelf edge based on habitat and prey variables measured along more than 20 transects, half of which did not have visual survey data available. The predicted hotspot matched the habitat used by tagged kittiwakes quite well – in fact the center of the hotspot used by kittiwakes was predicted by five transects – all of which lacked visual survey data. These transects without visual survey data were not included when the statistical model was generated, yet they did equally well in predicting the foraging kernels of each predator species.

Our results suggest a consistency in the fundamental relationships between predator distributions and within-patch measures of their prey base, regardless of temporal or spatial scales. For example, the foraging arena available (≤100 km) to central place foragers at each island showed no significant effect on the goodness of the models’ fit to the data for each predator species, despite there being known differences in the composition of the animals’ diets at each island [47], [80]. Similarly, year was not included as a factor in the model, yet the relationship between the predictor variables and the distribution predators was not significantly different between years. Finally, despite the fact that nighttime represents only about six to eight hours of each day in the Bering Sea during our study period, this is an important time period for predators in this system [27]. Our sampling, particularly in 2009, was designed to reflect that, with the result that about 40% of all transects did not have predator data available. Yet, prey and environmental data from these nighttime transects did just as well in predicting predators. In both years, during both day and night, and in all island foraging arenas, individual predators of each species always used the same “rules” to choose their foraging habitat, indicating that there are specific variables that make prey “suitable” for them.

We show that the fine-scale spatial distribution of prey is critical to how predators perceive the suitability of their food supply and the mechanisms they use to exploit it, regardless of time of day, sampling year, or source colony. These distributional characteristics had limited relationships with transect-averaged (areal) prey biomass density or prey abundance. For example, there was no significant relationship between prey areal biomass density or prey abundance on a transect and the local density of prey within a patch for either pollock or squid. Only the number of pollock patches detected had a clear relationship to a measure of integrated biomass (Figure 2a), showing a threshold effect for patch formation that is roughly ten times the biomass of a median pollock patch or 100 times the biomass of the minimum biomass of a pollock patch. However, the number of pollock patches per transect was not important in the prediction of any predator. The relationship between pollock patch numbers and integrated biomass could be used to relate existing data sets of juvenile pollock biomass to measures of the degree of pollock patchiness though not the *local* density of those patches, which we show is the critical measurement for predicting predator habitat use.

There was a strong fit between the model predictions and the habitat used by tagged predators (Figure 3, Table 1), however, the models tended to over-predict, on average, the number of predators that should use a given area. The models predicting observed predator distributions were based on all individuals of a species whereas the tagging data used to test these models included only breeding individuals for birds and only breeding females for fur seals. It is not surprising, then, that the survey data for predator densities and thus models based on the density of all predators consistently over-predicted the habitat use by tagged animals. The over-prediction of predators from the statistical models, however, was not uniform over the study area, an indication of the location specific costs and benefits for predators when they are functioning as central place foragers. The effect of colony location on the relative value of habitat was clear (Figure 4c). Even modestly good habitat, as predicated by our model, was used heavily if it was in very close proximity to an island (fur seals: <50 km, murres and kittiwakes: <25 km). At mid ranges (fur seals: 50–150 km, murres: 25–75 km, kittiwakes: 25–100 km) habitat was used as predicted. Only the habit characterized by the model as the very best was utilized by these predators as range from the island increased further. To model habitat use by these central place foragers, a distance from island weighting function must be applied for each species. For all three species, this function was different for Bogoslof Island than for each of the Pribilof Islands, with predators feeding exclusively close to Bogoslof Island while animals at the Pribilofs covered more extensive ranges. This may be due to differences in inter- and inter-specific competition as a function of colony size [81], [82] or the proximity of each island to oceanic habitat [83].

We conclude that when prey are distributed in discrete aggregations, as both juvenile walleye pollock and euphausiids were in this system, analysis needs to be done on the patch level rather than on an arbitrarily defined grid in order to observe strong coherence between predators and their prey [19], [21], [24]. This highlights the importance of quantifying prey suitability [sensu 2], a measure that is defined from the predator’s point of view rather than the researcher’s, when looking for predator-prey relationships [19], [24], [76]. Our results, coupled with predictions from foraging theory, suggest that prey distributional characteristics are the causal, driving forces for the distributions of northern fur seals, thick-billed murres, and black-legged kittiwakes. Descriptions of direct relationships, rather than proxies, allows the relationships we observed between specific predators and prey to be more easily generalized to other geographic areas and other predators. Further, at least over the range of values measured here, these models can be employed in a dynamic, predictive capacity in a changing environment [29]. This is critical in places such as the Bering Sea where the biology of the system is rapidly showing effects of climate change [84].

The lack of expected coherence between predators and prey in marine systems has generated a number of new hypotheses to explain observed mismatches [18], [85] and a general belief that “traditional foraging models do not adequately describe resource acquisition in marine environments” [15]. For example, Fauchauld [18] suggested that behaviorally generated spatial patterns such as schooling of prey and local enhancement of predators account for the mismatch, and thus, overlap between predators and prey should not be expected. Here, we show that incorporating spatial aggregation into the description of a prey field can reveal overlap between predators and prey that could not when using averaged prey concentrations, areal biomass densities, or the variance in these averaged measures. The consistent importance of prey spatial distribution to the habitat use of three co-occurring predator species with different constraints indicates the importance of spatial aggregations in determining the foraging of predators in the southeastern Bering Sea. Coupled with previous work from other high latitude systems [86] and subtropical ecosystems [87], our results indicate that predator-prey relationships in pelagic marine ecosystems may generally be regulated by patchiness.
